# Half-Yearly Report of Cases in Midwifery, Which Have Occurred in the Northern District of the London and Southwark Midwifery Institution

**Published:** 1829-08

**Authors:** C. Waller

**Affiliations:** Consulting Accoucheur to the above Institution, and Lecturer on Midwifery at the Medical School, 58, Aldersgate street.


### MIDWIFERY.

*Half-yearly Report of Cases in Midwifery, which have occurred in the Northern District of the London and Southwark Midwifery Institution.* By C. Waller, Esq. Consulting Accoucheur to the above Institution, and Lecturer on Midwifery at the Medical School, 58, Aldersgate street.

| 1829. | No. Women delivered. | Sex of Children: Males. | Females. | Born alive. | Stillborn. | Presentation. |
|---|---|---|---|---|---|---|
| January .. | 29 | 14 | 15 | 26 | 3 | 28 Natural<br>1 Face |
| February .. | 22 | 12 | 10 | 21 | 1 | Natural |
| March .... | 36 | 17 | 19 | 35 | 1 | 35 Natural<br>1 Placenta and Back |
| April .... | 34 | 14 | 20 | 30 | 4 | 32 Natural<br>1 Foot |
| May...... | 34 | 25 | 9 | 31 | 3 | Natural |
| June...... | 27 | 13 | 14 | 25 | 2 | 26 Natural<br>1 Foot |
| Total .... | 182 | 95 | 87 | 168 | 14 | |

Remarks.—Several of the children reported as stillborn have been premature. Nothing particular occurred, excepting in one case, where the patient was advanced a little beyond the sixth month of pregnancy. I was summoned to her in consequence of a sudden gush of blood following the discharge of the waters. On examination, I found the placenta attached to rather more than one half the circumference of the os uteri. The hemorrhage, in consequence of the tonic contraction of the uterus, was exceedingly trifling: in fact, there was no more discharge than there frequently is in a natural labour. The pains just then a little flagging, but still, during their intervals, the child was closely embraced by the womb. The presentation could not be distinctly ascertained at first. After a short period the pains increased, pushing down the placenta first, and the child afterwards, which, although a presentation of the back, was expelled double with tolerable ease; the uterus all this time retaining its contraction so firmly that the bleeding did not return; which, of course, rendered it quite unnecessary manually to interfere.

In both of the footling presentations the children perished. In one, profuse hemorrhage preceded the birth of the child, which continued notwithstanding the use of cold and friction. I therefore determined upon emptying the womb, and, on examination, found the vagina filled with coagula, in the midst of which was a foot, which was secured and easily brought down; when the hemorrhage instantly ceased. In consequence of a contracted brim, some little difficulty was experienced in bringing the head through this part of the pelvis, which was, perhaps, partly the cause of the infant's death. This patient had a good getting-up, though she remained faint for some time after delivery.

In the other case the female was suddenly delivered, the body of the child being born before she sent for her medical attendant. Owing to this circumstance, the head was detained in the vagina, the circulation through the cord interrupted, and of course the infant died.

In one instance, hemorrhage to a very considerable extent occurred after the expulsion of the placenta. The patient, a young woman, ætatis twenty, was delivered of a very large stillborn child, after a protracted labour: the placenta was expelled naturally, but the quantity of blood lost afterwards was very great, the bed being literally soaked through. No effect, however, was produced upon this patient's constitution by it, her pulse remaining steady and her countenance unchanged.

In another case, the hemorrhage took place between the birth of the child and the expulsion of the placenta. The patient's age was twenty-three, and she had just been delivered of her third child, after a slow labour, the uterus, till just at the termination of it, acting very feebly. After the birth of the child she seemed very comfortable, and, on placing my hand upon the abdomen, the uterus was felt pretty firm, though rather larger than usual. In a few minutes she became excessively faint, and, on examination, I found that blood was coming away in large quantities. The placenta was lying in the upper part of the vagina, and therefore, after employing cold and friction, I removed it, when the discharge abated; but there was a very considerable draining for some hours afterwards, so much as to produce repeated attacks of syncope.

In a third case, the hemorrhage occurred immediately after the birth of the placenta, the uterus remaining flabby for some time. The application of cold, with friction, at length arrested it, though not before the patient had been brought very low. The next day, on her being moved, the discharge recurred to a considerable extent. She is now suffering severely from headach and diarrhœa, the consequences of the hemorrhage.

A very firm and pretty general adhesion of the placenta occurred in one case: owing to the powerful action of the womb, no hemorrhage occurred, but the operation of extraction was by this circumstance rendered extremely difficult. The patient recovered afterwards, without an unpleasant symptom.

The face presentation happened to a patient whose pelvis was of good size, and where there was plenty of secretion, and therefore no great difficulty was experienced, although the labour was, of necessity, rendered more tedious and severe than under ordinary circumstances The child's face was very much tumefied, but regained its natural appearance after a few days.

A fatal case of abdominal inflammation has taken place within the last six months, after a very natural labour: as, however, I did not see this patient, I cannot enter into the detail of either symptoms or treatment.

In one individual the use of the long forceps was required, in consequence of the extraordinary size of the child. This patient had been in labour for two days, though the pains were not very urgent; the liquor amnii had unfortunately been discharged at the commencement of the labour. I succeeded, with some difficulty, in delivering the head, and at this period the child was alive; but, owing to the immense breadth of the shoulders, they could not be extricated in time to preserve its life: the united efforts of Mr. Doubleday and myself were required for a considerable time before we could accomplish our object. The weight of the infant, including the flannel in which it was wrapped, was twelve pounds eleven ounces. The female had a remarkably good getting-up.

In several instances, patients have complained of severe abdominal pains, somewhat simulating inflammation; pressure considerably aggravating their distress. The state of the countenance, the pulse, and the tongue, however, not being indicative of active inflammation, I prescribed calomel and opium, with the most marked success, in almost every case.

The induction of premature labour was required in one case, in consequence of great deformity of the pelvis. This female had been pregnant eleven times previously; she had three times been allowed to proceed to the full period, and each time it was found necessary to open the child's head; the remaining eight times, labour had been brought on at the seventh month, but none of the children survived long. I this time punctured the membranes on the 8th of June; on the 10th, slight labour-pains commenced; and on the 11th, early in the morning, the fœtus was expelled. This patient had been deceived in her reckoning; for, although she stated herself to have completed the seventh month, it was evidently not more than a six months' child. The fœtus was born living, but it never breathed.

Several interesting cases of false pains have occurred: in one female they were very violent, and resisted every means employed for their relief, so that I considered myself justified in rupturing the membranes, and thus hastening the parturient process, the parts of generation appearing to be well disposed for labour.

A considerable tumor formed on the head of one child, at the junction of the occipital with the parietal bone. According to the opinion of the late Dr. Baillie, these tumors are produced by extravasated blood, in consequence of one or more of the vessels of the scalp being lacerated at the time of the passage of the child's head. I have twice had an opportunity of seeing the contents of these swellings, and I found (as far as I could judge from these two cases,) that Dr. B.'s opinion was correct. They require little or no treatment. I usually order a Liq. Ammon. acet. lotion, with a view of increasing the process of absorption; but am by no means prepared to assert, that they would not disappear as soon without it.

*93, Bartholomew Close; July* 1829.

